# Pluripotent Human embryonic stem cell derived neural lineages for in vitro modelling of enterovirus 71 infection and therapy

**DOI:** 10.1186/s12985-015-0454-6

**Published:** 2016-01-06

**Authors:** May Shin Yap, Yin Quan Tang, Yin Yeo, Wei Ling Lim, Lee Wei Lim, Kuan Onn Tan, Mark Richards, Iekhsan Othman, Chit Laa Poh, Boon Chin Heng

**Affiliations:** Department of Biological Sciences, Faculty of Science & Technology, Sunway University, No. 5 Jalan Universiti, Bandar Sunway, 47500, Selangor Darul Ehsan Malaysia; The University of Hong Kong, Pokfulam, Hong Kong; School of Chemical & Life Sciences, Nanyang Polytechnic, 180 Ang Mo Kio Avenue 8, Singapore, 569830 Singapore; Jeffrey Cheah School of Medicine, Monash University Malaysia, Jalan Lagoon Selatan, 47500 Bandar Sunway, Selangor Darul Ehsan Malaysia

**Keywords:** EV71, HFMD, Infectious diseases, Neurons, Viral

## Abstract

**Background:**

The incidence of neurological complications and fatalities associated with Hand, Foot & Mouth disease has increased over recent years, due to emergence of newly-evolved strains of Enterovirus 71 (EV71). In the search for new antiviral therapeutics against EV71, accurate and sensitive in vitro cellular models for preliminary studies of EV71 pathogenesis is an essential prerequisite, before progressing to expensive and time-consuming live animal studies and clinical trials.

**Methods:**

This study thus investigated whether neural lineages derived from pluripotent human embryonic stem cells (hESC) can fulfil this purpose. EV71 infection of hESC-derived neural stem cells (NSC) and mature neurons (MN) was carried out in vitro, in comparison with RD and SH-SY5Y cell lines.

**Results:**

Upon assessment of post-infection survivability and EV71 production by the various types, it was observed that NSC were significantly more susceptible to EV71 infection compared to MN, RD (rhabdomyosarcoma) and SH-SY5Y cells, which was consistent with previous studies on mice. The SP81 peptide had significantly greater inhibitory effect on EV71 production by NSC and MN compared to the cancer-derived RD and SH-SY5Y cell lines.

**Conclusions:**

Hence, this study demonstrates that hESC-derived neural lineages can be utilized as in vitro models for studying EV71 pathogenesis and for screening of antiviral therapeutics.

## Background

Enterovirus 71 (EV71), the causative agent of Hand, Foot & Mouth disease (HFMD), is a single-stranded positive-sense RNA virus [1]. The clinical symptoms of HFMD is often mild, manifested by fever with papulovesicular rash on the soles and palms [2]. However, in recent years, new virulent strains of EV71 have evolved, with the potential to cause severe neurological complications and even fatalities in young children below six years of age [3, 4]. Currently, effective treatment modalities against such new strains of EV71 is lacking, and there is therefore an urgent and dire need to develop new antiviral therapeutics against EV71.

In the research and development pipeline, accurate and sensitive in vitro cellular models for investigating EV71 pathogenesis are of paramount importance in the preliminary screening and testing of newly-developed antiviral agents, before progressing to expensive and time-consuming in vivo animal studies, followed by clinical trials in human patients. Currently, various immortalized cell lines derived from cancers such as RD and Vero cells are widely utilized for in vitro studies of EV71 [5, 6]. RD cells are derived from human rhabdomyosarcoma [7], while Vero cells are derived from the kidney epithelium of African green monkeys [8]. There are some deficiencies in utilizing these cells for in vitro modelling of EV71 pathogenesis. In particular, there are inherent genetic abnormalities and karyotypic instability of these cells due to their neoplastic origin [9], which would poorly reflect normal human physiology in vivo. It is imperative for in vitro cellular models to closely mimic physiological conditions so as to provide a realistic picture of in vivo cellular interactions with infectious agents such as EV71.

Another major deficiency is that commonly-utilized cell lines for studying EV71 such as RD and Vero cells do not express the neural phenotype required to model neurological complications associated with HMFD, which often lead to fatalities in young children [3, 4]. For example, mature neurons of the central nervous system are mitotically quiescent and have unique electrophysiological properties not displayed by fast-proliferating RD and Vero cells. One solution may be to utilize neural tissues derived from laboratory animals or explanted from human cadavers, abortuses [10] and discarded pathological waste samples from brain and spinal cord surgery. Nevertheless, these alternative sources of neural tissues for in vitro studies also have their limitations. In particular, neurons from animal species may exhibit different electrophysiological properties to human neurons [11], and there is often scarce supply of neural tissues obtained from human cadavers, abortuses and discarded surgical waste, in addition to much inter-batch variability depending on the medical condition and age of the human donors. Moreover, it must be remembered that the proliferative capacity and in vitro lifespan of primary neural tissue cultures are also very much limited.

These limitations and deficiencies may be overcome by utilizing human neural lineages differentiated from human embryonic stem cells (hESCs) [12, 13], which are isolated from blastocyst-stage embryos [14, 15]. Unlike immortalized cancer-derived cell lines such as RD and Vero cells, most hESCs and their differentiated neural progenies are karyotypically stable and genetically normal [16, 17]. Compared to animal-derived neural explants, hESC-derived neural lineages are more representative of human central nervous system (CNS) physiology and may more accurately recapitulate the in vivo interaction between EV71 and the human CNS during pathogenesis, hence overcoming the problem of species-specificity. Due to the unlimited proliferative and self-renewal capacity of hESCs [14, 15], the problems of scarce supply, short lifespan and senescence associated with primary explanted human neural tissue cultures can therefore be avoided by obtaining neural lineages from hESCs. Moreover, efficient in vitro differentiation protocols for deriving neural lineages from hESCs have now been developed [18, 19], and various studies have also validated the excellent neural phenotype and electrophysiological properties displayed by these cells [20, 21].

All these advantages of hESC-derived neural lineages for in vitro studies have in turn led to their increasing adoption by neuroscience researchers for pathological modelling of neurodegenerative diseases, toxicology assessment, and drug screening [22]. However, the potential use of these cells for in vitro modelling of the pathogenesis of infectious diseases targeting the nervous system, such as EV71, has been much less studied.

This study will utilize EV71 strain 41, which was originally isolated from a fatal case of encephalitis during a major HMFD outbreak in Singapore in the year 2000 [23]. Previously, this particular EV71 strain was studied with AG129 strain mice that lacked alpha/beta-interferon and gamma-interferon receptor genes [24]. The objective of this study is to investigate EV71 infection of hESC-derived mature neurons and neural stem cells in vitro, in comparison with RD cells that is commonly-utilized for EV71 studies [5], as well as with a neuroblastoma-derived cell line SH-SY5Y [25]. Cell survivability after infection and EV71 replication will be assessed and compared amongst these four different cell types. Additionally, the inhibitory effect of SP81 peptide on EV71 replication, which was reported in a previous study [26], will also be compared amongst the four different cell types. The results obtained will be used to evaluate whether hESC-derived mature neurons and neural stem cells are suitable in vitro cellular models for studying EV71 pathogenesis.

## Methods

### Cells, viruses, reagents, culture media, and other consumables

The hESC line WA01 was purchased from Wicell Institute Inc. (Madison, WI, USA). RD cells and SH-SY5Y cells were purchased from the American Type Culture Collection (ATCC, Manassas, VA, USA). Unless otherwise stated, all chemical reagents (analytical grade) were purchased from Sigma-Aldrich Inc. (St. Louis, MO, USA), all plastic labware consumables were purchased from Becton-Dickinson Inc. (Franklin Lakes, NJ, USA), and all cell-culture media, supplements, coatings, serum, and serum supplements were purchased from Life Technologies Inc. (Carlsbad, CA, USA). Stocks of EV71 strain 41 (5865/SIN/000009) (GenBank accession number: AF316321) were obtained through propagation in RD cells with DMEM supplemented with 2 % FBS. SP81 peptide based on the VP1 capsid protein of EV71 was synthesized by Mimotopes Pty Ltd. (Clayton Victoria, Australia), with a 95 % HPLC purity grade. This peptide was reconstituted in 100 % dimethyl sulfoxide (DMSO) and stored at −80 °C, prior to being diluted and utilized for experimental study.

### Cell culture and expansion

The hESC line WA01 was routinely cultured on Geltrex™ (Cat No. A15696-01, Life Technologies Inc.) pre-coated 6-well culture plates with mTeSR1™ medium (Cat No. 05850, Stem Cell Technologies Inc., Vancouver, BC, Canada). Upon reaching confluence after 5 to 7 days of culture, serial passage was carried out with enzymatic dissociation of colonies into cellular clumps with 1 mg/ml of collagenase. The RD cells and SH-SY5Y cells were routinely cultured in T75 cell culture flasks in culture milieu comprising of Dulbecco’s Minimum Essential medium (DMEM), supplemented with 10 % (v/v) fetal bovine serum (FBS) and 1 % (v/v) penicillin streptomycin antibiotic solution. When cultures became confluent after 5–7 days of culture, serial passage at a split ratio of 1:4 to 1:5 was carried out through enzymatic dissociation with 0.05 % Trypsin–EDTA solution. All cell cultures were maintained within a 5 % CO_2_ incubator at 37 °C.

### Induction of human embryonic stem cells into neural stem cells

Confluent hESC cultures were dissociated into cellular clumps with 1 mg/ml collagenase, washed once with phosphate buffered saline (PBS) through centrifugation and then digested into a single cell suspension through treatment with StemPro™ Accutase Cell Dissociation Reagent (Cat. no. A11105, Life Technologies Inc.), for 5 to 10 min followed with gentle pipetting. The dissociated hESC were then reconstituted in mTeSR1™ medium supplemented with 10 μM ROCK Inhibitor Y27632 (Cat. no. Y0503, Sigma-Aldrich Inc.), and plated into 6-well culture plates pre-coated with Geltrex™, at a density of 2.5 × 10^5^–3 × 10^5^ cells per well. The following day, induction of hESC into NSC was carried out by replacing the culture milieu with Neurobasal™ medium (Cat No. 21103-049, Life Technologies Inc.) supplemented with 2 % (v/v) Neural Induction Supplement™ (Cat No. A16477-01, Life Technologies Inc.) and 1 %(v/v) penicillin-streptomycin antibiotic solution. Cultures became confluent after 6 to 7 days of culture in a 5 % CO_2_ incubator set at 37 °C, and were serially passaged onto Geltrex™ pre-coated 6-well plates with StemPro Accutase Cell Dissociation Reagent™, utilizing similar aforementioned seeding densities. NSC from the third passage onwards were utilized for differentiation into mature neurons, as well as for further experiments.

### Induction of neural stem cells into mature neurons

NSC cultures were dissociated into a single cell suspension with StemPro™ Accutase Cell Dissociation Reagent, washed once in PBS through centrifugation, and then seeded on 6 or 24-well culture plates pre-coated with Poly-L-Ornithine (10 μg/ml) and Laminin (20 μg/ml) at a seeding density of 2.5–5 × 10^4^ cells/cm^2^, in complete StemPro™ NSC SFM (serum-free medium) comprised of KnockOut™ D-MEM/F-12 (Cat. No. 12660, Life Technologies Inc.), supplemented with 2 % (v/v) StemPro™ Neural Supplement (Cat. No. A1050801, Life Technologies Inc.), 20 ng/ml EGF (Cat. No. PHG0314, Life Technologies Inc.), 20 ng/nl bFGF (Cat. No. PHG0024, Life Technologies Inc.), 2 mM (L-glutamine), and 1 % (v/v) penicillin-streptomycin antibiotic solution. After 2 days of culture in StemProTM™ NSC SFM, the medium was changed to neural differentiation medium, comprised of Neurobasal™ medium supplemented with 2 % (v/v) B-27™ Serum-Free Supplement, 2 mM L-glutamine and 1 % (v/v) penicillin-streptomycin antibiotic solution. Fresh neural differentiation medium was replaced every 3–4 days. From day 7 onwards, 0.5 mM of dibutyryl cAMP (Cat. No. D0627, Sigma-Aldrich Inc.) was added to the neural differentiation medium daily, to expedite differentiation into mature neurons (MN). After 10 days of differentiation culture, the mature neurons obtained were utilized for further experiments.

### Differentiation of SH-SY5Y cells

SH-SY5Y cells were seeded onto 6 or 24-well culture plates at a density of 3 × 10^3^ to 1 × 10^5^ cells/cm^2^ with DMEM supplemented with 10 % (v/v) FBS and 1 % (v/v) penicillin streptomycin antibiotic solution. The following day, serum-containing medium was replaced with Neurobasal™ medium supplemented with 2 % (v/v) B-27™ Serum-Free Supplement, 2 mM L-glutamine, 10 μM all-trans-retinoic acid, and 1 % (v/v) penicillin-streptomycin antibiotic solution, to promote differentiation and neuronal phenotype. SH-SY5Y cells were cultured for 10 days under differentiation conditions, prior to being utilized for further experiments.

### Immunocytochemistry for detection of neural markers

After neural induction and differentiation, NSC and MN were fixed with 4 % (v/v) paraformaldehyde for 15 min, permeabilized with 0.1 % Triton X-100 for 10 min, and then blocked in PBS supplemented with 10 % (v/v) FBS for 2 h. The fixed cells were then incubated for 2 h at room temperature with the appropriate primary antibodies: anti-doublecortin antibody (Cat. No. sc-271390, Santa Cruz Biotechnology Inc., Santa Cruz, CA, USA), and anti-nestin antibody (Cat. No. sc-23927, Santa Cruz Biotechnology Inc., Santa Cruz, CA, USA) diluted in 1 % (w/v) BSA/PBS at appropriate concentrations. The cells were then washed in 1 % BSA/PBS and incubated in the dark with the appropriate secondary antibodies: Goat anti-Mouse IgG FITC-conjugated (Cat. No. sc-2010, Santa Cruz Biotechnology Inc., Santa Cruz, CA, USA) and Goat anti-Mouse IgG PE-conjugated (Cat. No. sc-358926, Santa Cruz Biotechnology Inc., Santa Cruz, CA, USA) for 2 h at room temperature. The secondary antibodies were diluted in 1 % BSA/PBS at appropriate concentrations. Following another wash with 1 % (w/v) BSA/PBS, the cell nuclei were stained with DAPI, prior to imaging under a Nikon Eclipse T*i*™ fluorescent microscope with the appropriate excitation/emission wavelength.

### qRT-PCR analysis of neural marker expression

Quantitative real-time RT-polymerase chain reaction (qRT-PCR) analysis of neural marker gene expression was carried out as described previously [27]. Briefly, RNA from undifferentiated hESC (WA01), NSC, MN, RD and SH-SY5Y cells were harvested using the InnuPREP™ RNA mini kit 86 (Cat No. 845-ks-1040050, Analytik Jena GmbH, Jena, Germany) with DNase treatment. cDNA was synthesized from the extracted RNA using the high capacity cDNA reverse transcriptase kit (Cat. No. 4374966, Applied Biosystems Inc, Carlsbad, CA, USA). qRT-PCR analysis was then performed with a Biorad C1000 Touch™ thermal cycler (Bio-Rad Laboratories, Hercules, CA, USA), utilizing SYBR Green™ Mastermix (Cat No. 4367659, Life Technologies Inc., Carlsbad, CA, USA) with appropriate custom-designed primers for early neural markers: Pax6, Musashi1 and βIII-tubulin, and mature neural markers: MAP2 (Microtuble Associated Protein 2), NCAM (Neural Cell Adhesion Molecule), NFM (Neural Filament Protein - Medium) and NSE (Neural Specific Enolase), as previously described [27]. The following amplification parameters were utilized for qRT-PCR: two minutes at 50 °C, 20 s at 95 °C, and 60 cycles of 1 s at 95 °C, followed by one minute at 60 °C. The relative Cycle Threshold (Ct) was determined and normalized against the endogenous GAPDH housekeeping gene. The fold change of each gene was compared against either undifferentiated hESC or NSC. All bar charts and graphs show standard deviations representing at least three experimental replicates.

### Infection of different cell types with EV71 and inhibition of viral replication with SP81 peptide

NSC, MN, RD and SH-SY5Y cells were seeded in 24 well-plates at a density of 4.0 X 10^5^ cells per well overnight. The following day, cell counts (hemocytometer) were performed on trypsin-dissociated cells from two to three representative wells for each cell type, because the cell number in each well would be expected to change due to proliferation and apoptosis during the seeding process. For each of the four different cell types, some of the wells were treated for 1 h with 100 μM of the SP81 peptide reconstituted in serum-free medium, while other wells were left untreated. The four different cell types were then infected with diluted stocks of EV71 in serum-free medium, at a constant Multiplicity of Infection (MOI) of 0.1. After adsorption for 1 h, the inoculum was removed, and the cells were washed twice with the serum-free medium, prior to replacement of the appropriate maintenance medium for each of the four different cell types. Altogether, there were three experimental groups for each different cell type: (i) untreated control, (ii) treated with EV71 only, and (iii) treated with both SP81 peptide and EV71. Cell culture supernatants were harvested at the 24-, 72- & 120-h post-infection timepoints, for all four different cell types and three treatment groups, for quantification of viral copy number via qRT-PCR assay.

### Assessment of cell survivability after infection with EV71, with and without pre-treatment with SP81 peptide

Cell survivability after exposure to the SP81 peptide and EV71, were assessed with the CellTiter-Blue™ cell viability assay (Promega Inc., Madison, WI, USA), according to the manufacturer’s instructions. This assay is based on change in fluorescent properties upon the reduction of resazurin (Alamar Blue) to resorufin by metabolically-active viable cells [28]. At the 24 h and 120 h timepoints, untreated cells and cells treated with SP81 and/or EV71, were washed three times in PBS, prior to the addition of 50 μl of CellTiter-Blue™ solution in 200 μl of culture media within each well of the 24-well culture plate. After incubation for 2 to 3 h at 37 °C within a 5 % CO2 incubator, 200 μl aliquots of the reaction mixture were transferred onto a fresh 96-well plate, and fluorescence readings were measured spectrophotometrically at an excitation/emission wavelength of 560/590 nm respectively, utilizing a Tecan infinite M200 Pro™ microplate reader (Tecan Inc., Maennedorf, Switzerland). The cell viability after exposure to SP81 peptide and/or EV71, was calculated as the ratio of fluorescence readings (560/590 nm) yielded by the treated and untreated wells, after correction for blank fluorescence reading of the reaction mixture incubated without cells for the same duration (2 to 3 h) at 37 °C.

### Reverse Transcription (RT) TaqMan Real-time PCR Assay for determination of virus copy number in cell culture supernatants

The primers and probe were designed according to Tan et al. [26]. The forward primer employed was 59-GAGCTCTATAGGAGATAGTGTGAGTAGGG-39, the reverse primer was 59-ATGACTGCTCACCTGCGTGTT-39 and the TaqMan™ probe used was 596-FAM-ACTTACCCA/ZEN/GGCCCTGCCAGCTCC lowa Black FQ-39 (Applied Biosystems Inc., Carlsbad, USA). The viral RNA samples were extracted from the collected cell culture supernatants (Days 1, 3 and 5 post-infection) using the QIAamp™ Viral RNA mini kit (QIAGEN, Hilden, Germany) according to the manufacturer’s instructions. The RT TaqMan real-time PCR assay was performed with the Biorad C1000 Touch™ thermal cycler (Bio-Rad Laboratories, Hercules, CA, USA) using TaqMan™ Fast virus 1-step master mix (Applied Biosystems Inc., Carlsbad, USA) with cDNA synthesis by reverse transcription for 5 min at 50 °C and subsequent amplification for 40 cycles at 95 °C for 3 s, 60 °C for 30 s.

### Statistical analysis of data

All bar charts and graphs show standard deviations representing at least three replicate measurements. Ether the Chi-squared test or Student’s *t*-test were carried out to determine whether observed differences were statistically significant between different experimental conditions (*P* < 0.05 was considered statistically significant).

## Results

### Induction of human embryonic stem cells into neural stem cells, followed by differentiation into mature neurons

**Fig. 1:**
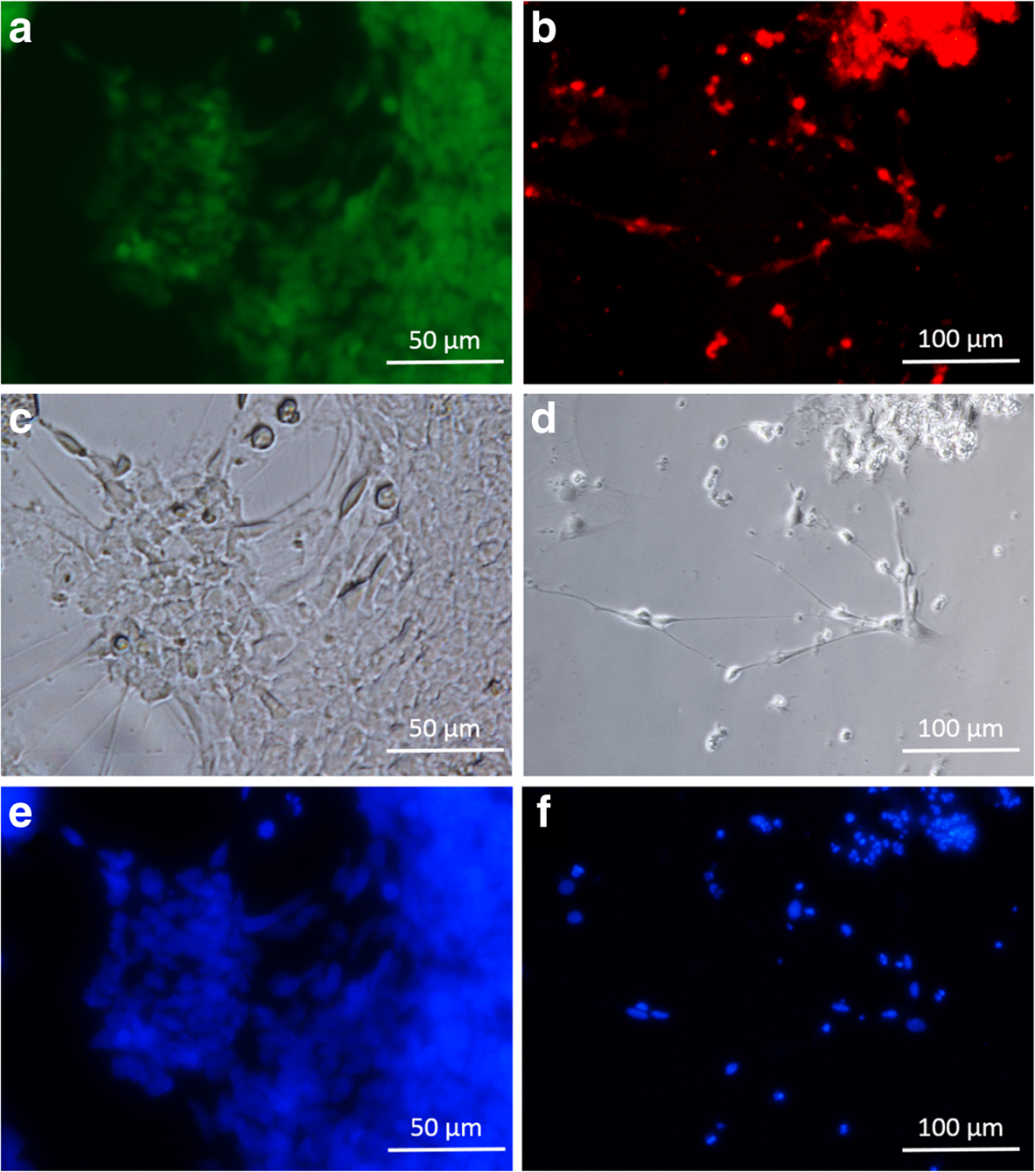
Immunostaining of (**a**) neural stem cells (NSC) for expression of Nestin, and (**b**) mature neurons (MN) for expression of Doublecortin. **c** and **d** are the corresponding phase-contrast light microscopy images of (**a**) and (**b**) respectively. **e** and **f** are the corresponding DAPI-stained images (nuclear DNA) of (**a**) and (**b**) respectively

**Fig. 2:**
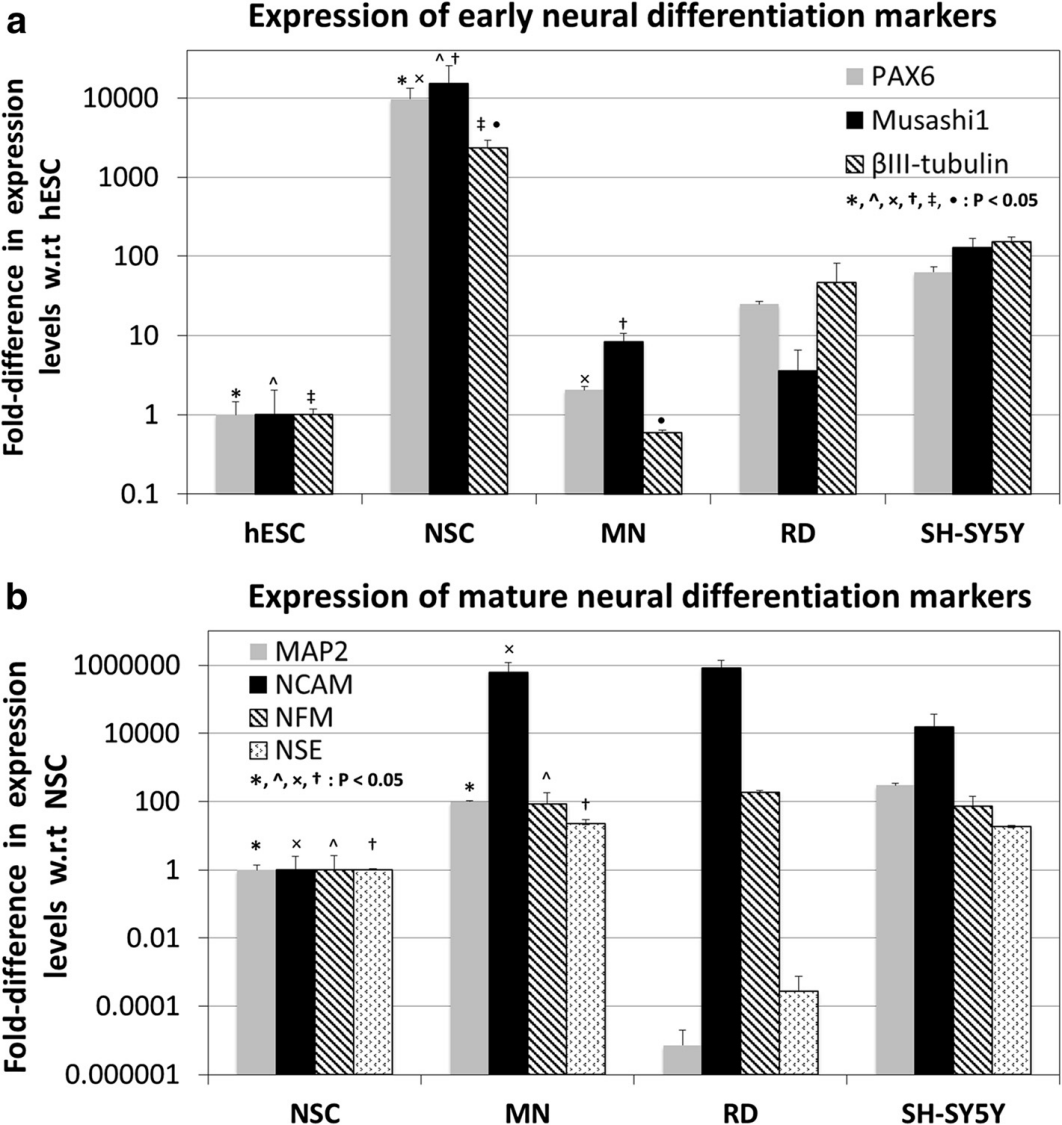
qRT-PCR analysis of the expression of (**a**) early neural differentiation markers, and (**b**) mature neural differentiation markers by hESC, NSC, MN, RD and SH-SY5Y cells. *, ^, ×, †, ‡, • : Significantly different at *P* < 0.05 (Student’s *t*-test)

Immunocytochemistry and qRT-PCR analysis of neural marker expression, was utilized to validate the NSC and MN populations obtained from hESC differentiation. As seen in the immunocytochemistry results presented in Fig. 1, there is positive expression of Nestin, a neural stem cell marker [29] by the NSC population derived from the hESC WA01 line in this study. Likewise, positive expression of Doublecortin, a mature neural marker [30], was also detected in the hESC-derived MN population. The qRT-PCR results (Fig. 2a) showed significant upregulation of early neural differentiation markers: PAX6, Musashi1 and βIII-tubulin [31–33] upon induction of hESC into NSC (9772.4, 15203.4 & 2329.0-folds respectively with respect to (w.r.t) hESC, *P* < 0.05), followed by a significant downregulation upon differentiation of NSC into MN (2.1, 8.3 & 0.6-folds respectively w.r.t hESC, *P* < 0.05). The RD and SH-SY5Y cells displayed much higher expression of all three early neural markers, as compared to undifferentiated hESC (after normalization with housekeeping gene GAPDH expression). The fold-differences in expression levels of PAX6, Musashi1 and βIII-tubulin by RD cells w.r.t. hESC were 25.0, 3.6 & 46.9 respectively, while the corresponding fold-differences in expression of these gene by SH-SY5Y cells were 62.8, 129.8 & 152.0 respectively. As seen in Fig. 2b, qRT-PCR analysis of mature neural marker expression: MAP2, NCAM, NFM & NSE [34–37] showed significant upregulation of these marker genes upon differentiation of NSC to MN (99.0, 624782.3, 86.5 & 22.0-folds respectively w.r.t. NSC, *P* < 0.05). SH-SY5Y cells displayed elevated expression of all four marker genes: MAP2, NCAM, NFM & NSE compared to NSC, after normalization with GAPDH (303.3, 15343.5, 72.6 & 18.5-folds w.r.t. NSC respectively). By contrast, RD cells displayed elevated expression of only NCAM and NFM compared to NSC (816211 and 190.5-folds w.r.t NSC respectively), while expression of MAP2 and NSE were much lower than that of NSC (7.2 X 10^−6^ and 3.0 X 10^−4^-folds w.r.t NSC respectively).

### Cell survivability after infection with EV71, with and without pre-treatment with SP81 peptide

**Fig. 3:**
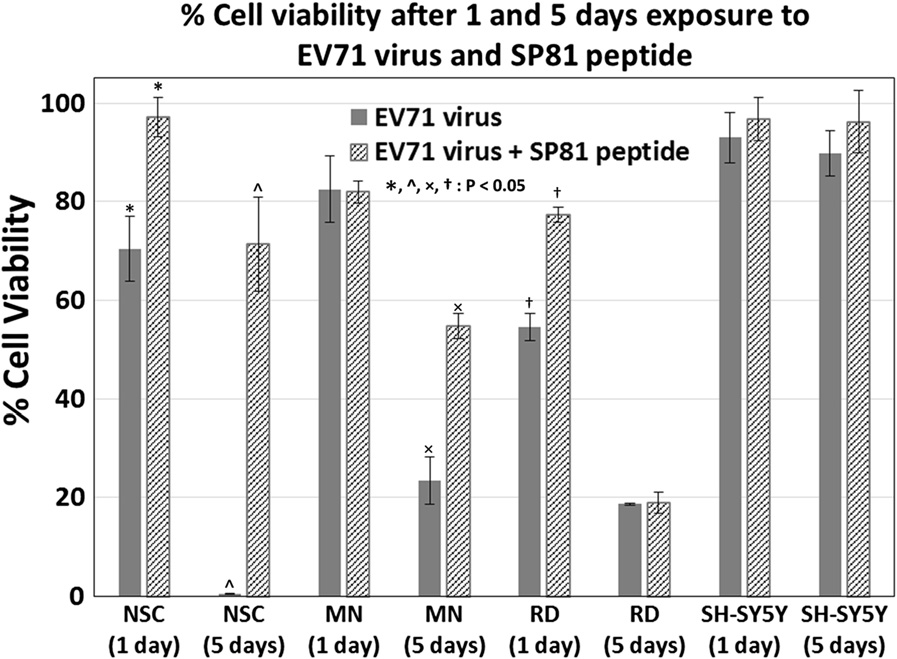
Viability of NSC, MN, RD and SH-SY5Y cells, after 1 and 5 days exposure to EV71, with or without prior treatment with SP81 peptide (CellTiter-Blue™ assay). *, ^, ×, † : Significantly different at *P* < 0.05 (Chi-squared test)

The CellTiter-Blue™ assay based on resazurin (Alamar Blue) was used to assess survivability of NSC, MN, RD and SH-SY5Y cells at 1 & 5 days after infection with EV71, with and without pre-treatment with SP81 peptide. The results (Fig. 3) showed that with the exception of SH-SY5Y cells that displayed minimal cell death after EV71 infection, all the other cell types (NSC, MN & RD cells) displayed substantial loss of viability upon infection with EV71. Moreover, it was observed that with the exception of SH-SY5Y cells, pre-treatment with the SP81 peptide had significant effects in improving survivability of all other cell types (NSC, MN & RD cells), at either or both the 1 & 5 days post-EV71 infection time-points. At the 1 day time-point, the viability of NSC, MN & RD cells, without SP81 peptide pre-treatment were 70.4 ± 6.6 %, 82.5 ± 6.8 % & 54.6 ± 2.8 % respectively, as opposed to 93.0 ± 5.1 % for SH-SY5Y cells. With SP81 peptide pre-treatment, the viability of NSC and RD cells at the 1 day post-infection time-point were significantly increased to 97.1 ± 4.1 % and 77.2 ± 1.5 % respectively (*P* < 0.05); whereas the corresponding viability of MN and SH-SY5Y cells were not significantly changed (82.0 ± 2.3 % and 96.7 ± 4.4 % respectively, *P* > 0.05) by SP81 peptide pre-treatment. At the 5 day time-point, the viability of NSC, MN & RD cells, without SP81 peptide pre-treatment were 0.5 ± 0.1 %, 23.4 ± 4.9 % & 18.7 ± 0.2 % respectively, as opposed to 89.8 ± 4.7 % for SH-SY5Y cells. With SP81 peptide pre-treatment, the viability of NSC and MN cells at the 5 days post-infection time-point were significantly increased to 71.3 ± 9.5 % and 54.7 ± 2.6 % respectively (*P* < 0.05); whereas the corresponding viability of RD and SH-SY5Y cells were not significantly changed (18.9 ± 2.2 % and 96.2 ± 6.3 % respectively, *P* > 0.05) by SP81 peptide pre-treatment.

### qRT-PCR analysis of EV71 replication in the various cell types, with and without pre-treatment with SP81 peptide

**Fig. 4:**
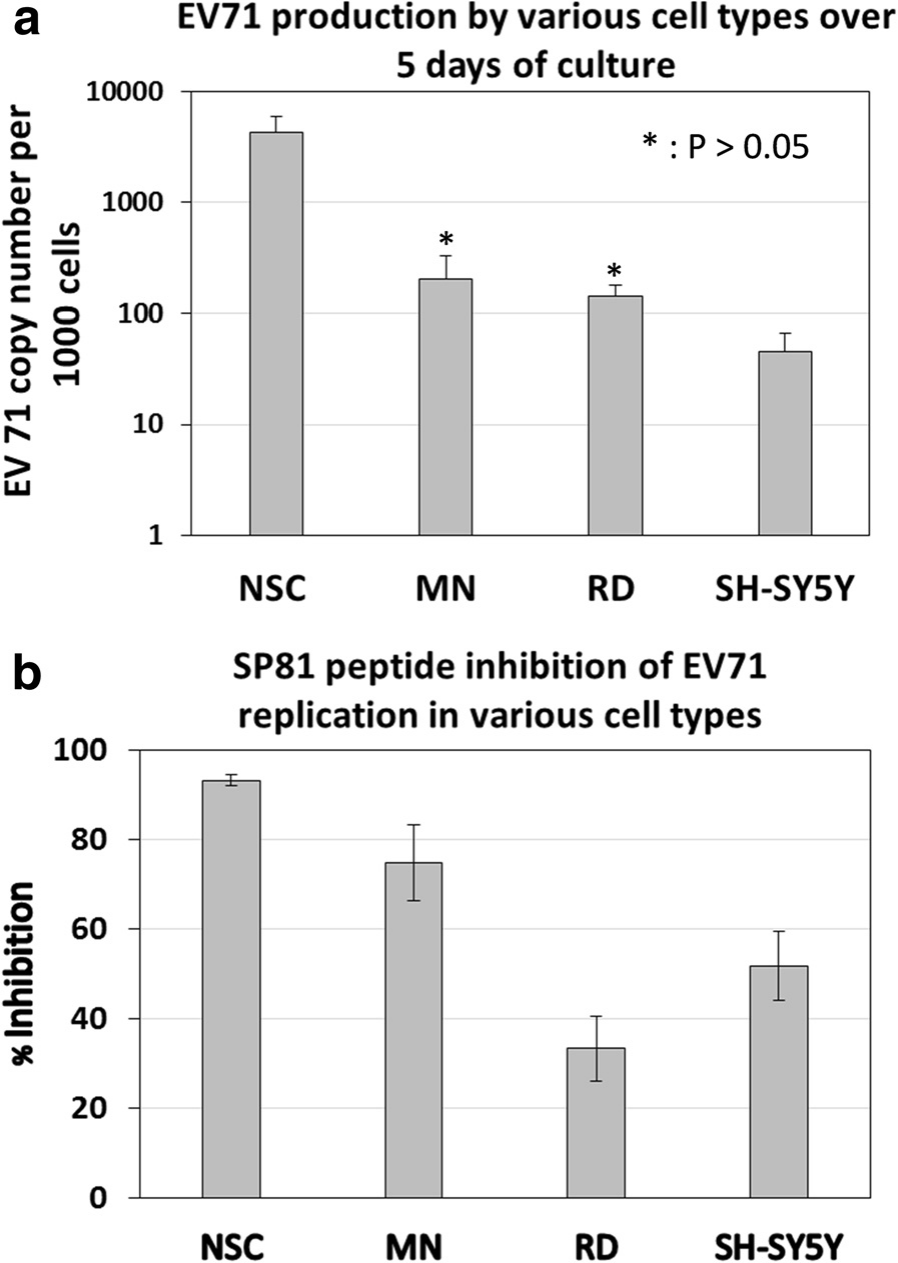
. qRT-PCR analysis of (**a**) EV71 production by NSC, MN, RD and SH-SY5Y cells over 5 days of culture. **b** SP81 peptide inhibition of EV71 replication in NSC, MN, RD and SH-SY5Y cells over 5 days of culture. Results of all experimental groups were significantly from each other, unless otherwise indicated. * : Not significantly different at *P* > 0.05 (Student’s *t*-test)

As seen in Fig. 4a, qRT-PCR analysis showed that the infected NSC yielded the highest level of EV71 replication, with a viral copy number of 4336.7 ± 1573.1 per 1000 infected cells. This was significantly higher than the corresponding values obtained for MN, RD and SH-SY5Y cells (205.1 ± 128.7, 142.6 ± 35.2 and 45.6 ± 20.5 respectively, *P* < 0.05). Upon pre-treatment with SP81 peptide, NSC exhibited the highest % inhibition of EV71 replication (computed as the percentage reduction in viral copy number) of 93.2 ± 1.2 %, which was significantly higher than the corresponding values obtained for MN, RD and SH-SY5Y cells (74.7 ± 8.4 %, 33.2 ± 7.3 % and 51.7 ± 7.6 % respectively, *P* < 0.05).

## Discussion

This study derived NSC and MN from undifferentiated hESC and utilized these cells for modelling EV71 pathogenesis in vitro, in comparison to cancer-derived RD and SH-SY5Y cell lines [7, 25]. Prior to utilizing these cells for in vitro studies with EV71, it was imperative to characterize and validate the neural phenotype of these cells with immunocytochemistry and qRT-PCR. Consistent with the scientific literature [29, 30], Nestin and Doublecortin were positively expressed by NSC and MN respectively (Fig. 1). The qRT-PCR data (Fig. 2a) showed strong upregulation of early neural differentiation markers (PAX6, Musashi1, & βIII-tubulin) upon induction of undifferentiated hESC into NSC, followed by a sharp downregulation of these genes upon further differentiation of NSC into MN, which is consistent with their known function [31–33]. By contrast, there was a strong upregulation of mature neural differentiation markers (MAP2, NCAM, NFM & NSE) during the transition of NSC to MN, which is again consistent with the scientific literature [34–37]. Hence, these data validated the neural phenotype of the MN and NSC populations.

The relatively high expression of both early and mature neural differentiation markers by SH-SY5Y cells is expected, given its origin from neuroblastoma [25], and that these cells have been subjected to neural differentiation for 10 days in the presence of retinoic acid [38]. Interestingly, it was found that RD cells of smooth muscle rhabdomyosarcoma origin [7] displayed relatively high expression of both early neural differentiation markers: (PAX6, Musashi1, & βIII-tubulin (Fig. 2a), as well as the mature neural differentiation markers NCAM and NSE (Fig. 2b). This appears to be consistent with previous studies that reported expression of neural markers by skeletal muscle tumors [39–41]. For example, Miettinen and Rapola [39] found that rhabmyosarcoma and rhabmyosarcoma-like tumors from a wide range of patients exhibited immunoreactivity to NFM protein, while Molenaar and Muntinghe [40] reported that the expression of NCAM and NFM isoforms were widely expressed by skeletal muscle tumors. Similarly, Soler et al. [41] also reported that Rhabmyosarcoma-derived cell lines displayed aberrant expression of various neural attachment proteins such as NCAM and N-Cadherin. This may suggest that RD cells could be suitable in some ways for modelling neurological complications associated with EV71 infection, since these cells also express various neural markers.

Subsequently, NSC and MN, together with RD and SH-SY5Y cells were utilized for in vitro studies with EV71. Assessment of cell viability after EV71 infection showed that SH-SY5Y cells exhibited the highest, and NSC the lowest post-infection survivability amongst the 4 different cell types. The observed much higher susceptibility of NSC to EV71 infection as compared to MN and SH-SY5Y cells (that have been differentiated with retinoic acid), is consistent with the study of Huang et al. [42], which showed that mice neural progenitor cells are much more susceptible to EV71 infection, as compared to mature neural lineages. Similar results were also reported by another study on mice [43], which showed that the closely-related Coxsackie virus of the genus Enterovirus, specifically targets fast-proliferating neural progenitor cells of the neonatal central nervous system. Hence, our in vitro data on human NSC and MN (derived from hESC), therefore validates the results of previous studies on the murine model [42, 43].

In the previous study of Tan et al. [26], the protective effects of a number of potential antiviral peptides against EV71 was assessed and compared; and it was shown that the SP40 peptide had a more potent antiviral effect against EV71, as compared to the SP81 peptide. Nevertheless, this study chose to examine SP81 instead of SP40, precisely because its lesser potency would enable a better assessment of the sensitivity of our in vitro cell culture model for screening antiviral agents. Pre-treatment with SP81 peptide seemed to exert an insignificant effect on the already high post-infection survivability of SH-SY5Y cells, in contrast to the sharp increase in post-infection survivability observed with NSC pre-treated with this peptide. Interestingly, it was observed that the SP81 peptide significantly improved the post-infection survival rates of NSC and RD cells on day 1, while there was no significant improvement in the case of MN. By contrast, 5 days after infection, it was observed that SP81 peptide had a significant protective effect on the post-infection survivability of MN. This is hypothesized to be due to differences in the mitotic rate of MN versus NSC and RD cells. Previous studies on the closely-related Coxsackie virus of the genus Enterovirus, have demonstrated that faster proliferating cells are much more susceptible to viral infection, as compared to mitotically quiescent cells [44, 45]. Because MN would be expected to be much less mitotically active compared to RD and NSC, EV71 infection of MN would presumably take place much more slowly. Hence, the protective effect of the SP81 peptide on MN survivability is not apparent on day 1, but became apparent after 5 days post-infection (Fig. 3).

qRT-PCR analysis of EV71 production by the various cell types over 5 days of culture (Fig. 4a) showed that NSC produced the highest yield of virus within the cell culture supernantant that was significantly higher than MN. Again, this is consistent with aforementioned previous studies in mice that demonstrated the greater susceptibility of neural progenitors to enterovirus compared to mature neural lineages [42, 43]. Rhoades et al. [46] hypothesized that neurotropic enteroviruses such as EV71 evolved to specifically target neural progenitors and stem cells (NPSCs) within the central nervous system, as this facilitates viral tropism and transmission. Because NPSCs actively proliferate during neonatal development, as well as occasionally during adult life, infection of NPSCs rather than mitotically-inactive mature neural lineages would thus expand the tropism of enteroviruses. Moreover, the ability of NPSCs to migrate as they differentiate and give rise to mature neural lineages would also maximize the transmission of enteroviruses throughout the entire central nervous system. Specific targeting of NPSCs by enterovirus might also be a strategy to establish persistent infection with sporadic reactivation whenever quiescent NPSCs are activated to give rise to mature neural lineages. Clinical symptoms of HFMD-associated neurological complications are more frequently manifested in young children and infants than mature adults [3, 4]. It is hypothesized that this could be due to more mitotically-active neural progenitors being present in their central nervous system compared to adults, which in turn makes them more susceptible to EV71 infection. Indeed, our results which showed higher EV71 copy number in NSC versus MN cells is consistent with this hypothesis.

Interestingly, it was observed that although the cell viability of SH-SY5Y cells was quite resistant to EV71 infection (Fig. 3), a substantial amount of virus was detected in the cell culture supernatant with qRT-PCR, even though this was still the lowest amongst the four different cell types (Fig. 4a). The results observed for SH-SY5Y cells are surprising considering that these cells are of human neural origin (neuroblastoma), have a relatively high mitotic rate, and display relatively enhanced expression of early/mature neural differentiation markers [25]. Apoptosis in mammalian cells is readily triggered by viral infection, which serves as a naturally-evolved host defense mechanism against pathogenic viruses [47]. Nevertheless, it must be remembered that the apoptotic machinery in cancer-derived immortalized cell lines like SH-SY5Y is highly-deficient, making these cells much more robust, and enabling them to maintain their viability in the face of severe environmental insults or stress factors that would normally trigger apoptosis in normal primary somatic cells of non-cancerous origin.

Subsequent analysis of the inhibitory effect of SP81 peptide on EV71 production by the four different cell types showed that NSC and MC exhibited significantly higher levels of inhibition compared to RD and SH-SY5Y cells (Fig. 4b). This would thus indicate that hESC-derived NSC and MN could provide a more sensitive in vitro model for screening of antiviral therapeutics, as compared to cancer-derived cells lines such as RD and SH-SY5Y cells.

## Conclusions

In conclusion, our study successfully replicated previous data on enteroviral infection of mice [42, 43] with in vitro cultured human NSC and MN derived from hESC. This thus demonstrates that hESC-derived NSC and MN can be utilized for in vitro modelling of the pathogenesis of neurotropic viruses such as EV71, as well as provide a more sensitive in vitro tool for preliminary screening of corresponding antiviral therapeutics, before progressing to expensive and time-consuming live animal studies and human clinical trials. Additionally, our findings on the greater susceptibility of NSC to EV71 infection compared to MN, may also shed some light on why young children are more prone to HFMD-associated neurological complications compared to adults [3, 4]; likely due to more mitotically-active neural progenitor cells being present in their central nervous system compared to adults.
